# The Antiproliferative Activity of Sclerotia of *Lignosus rhinocerus* (Tiger Milk Mushroom)

**DOI:** 10.1155/2012/697603

**Published:** 2012-02-13

**Authors:** M. L. Lee, N. H. Tan, S. Y. Fung, C. S. Tan, S. T. Ng

**Affiliations:** ^1^CENAR and Department of Molecular Medicine, Faculty of Medicine, University of Malaya, 50603 Kuala Lumpur, Malaysia; ^2^Biotechnology Research Centre, MARDI, P.O. Box 12301, 50744 Kuala Lumpur, Malaysia; ^3^Ligno Biotech Sdn Bhd, Taman Perindustrian Balakong Jaya 2, Selangor, 43300 Balakong Jaya, Malaysia

## Abstract

*Lignosus rhinocerus*, the tiger milk mushroom, is one of the most important medicinal mushrooms used by the indigenous people of Southeast Asia and China. It has been used to treat breast cancer. A cold water extract (LR-CW) prepared from the sclerotia of *L. rhinocerus* cultivar was found to exhibit antiproliferative activity against human breast carcinoma (MCF-7) and human lung carcinoma (A549), with IC_50_ of 96.7 **μ**g/mL and 466.7 **μ**g/mL, respectively. In comparison, LR-CW did not show significant cytotoxicity against the two corresponding human normal cells, 184B5 (human breast cell) and NL 20 (human lung cell). DNA fragmentation studies suggested that the cytotoxic action of LR-CW against cancer cells is mediated by apoptosis. Sephadex G-50 gel filtration fractionation of LR-CW yielded a high-molecular-weight and a low-molecular-weight fraction. The high-molecular-weight fraction contains mainly carbohydrate (68.7%) and small amount of protein (3.6%), whereas the low-molecular-weight fraction contains 31% carbohydrate and was devoid of protein. Only the high-molecular-weight fraction exhibited antiproliferative activity against cancer cells, with IC_50_ of 70.0 **μ**g/mL and 76.7 **μ**g/mL, respectively. Thus, the cytotoxic action of the LR-CW is due to the high-molecular-weight fraction, either the proteins or protein-carbohydrate complex.

## 1. Introduction

Mushroom has been consumed by many societies throughout the world due to its tastiness, high nutritional values, and pharmacological properties [1, 2]. It is well established that mushroom extracts contain a wide variety of compounds such as polysaccharides, protein, fibre, lectins, and polyphenols, each of which may have its own pharmacological effects [3]. Many mushrooms or their extracts can be used as therapeutic agents, and they are generally known as medicinal mushrooms.

*Lignosus rhinocerus*, the tiger milk mushroom, belongs to the Polyporaceae family and is one of the most important medicinal mushrooms used by natives in Southeast Asia and southern China. In Malaysia, the mushroom is also known locally as “*cendawan susu rimau*”—literary “mushroom of tiger's milk.” It is widely used by the indigenous communities in peninsular Malaysia to treat a variety of diseases, including breast cancer, fever, cough, asthma, food poisoning, and as a general tonic. The mushroom indeed is the most popular medicinal mushrooms used by the Malaysia indigenous populations [4]. In China, *L. rhinocerus *sclerotium is an expensive folk medicine used by traditional Chinese physicians to treat liver cancer, chronic hepatitis, and gastric ulcers [5]. The sclerotium of *L. rhinocerus *is the part with medicinal value. There are, however, very few studies on the pharmacological activites of the mushroom due mainly to its limited supply. The mushroom proved very difficult to cultivate and, until recently, was only available by collection from the jungle. Recently, Tan [6] reported successful cultivation of the mushroom in agar, solid, and spawn medium with good yield, thus making it possible to obtain large quantity for investigation and therapeutic purpose.

Lai et al. [7] was the first to investigate the antiproliferative effects of the sclerotial polysaccharides of the mushroom. The mushroom they used for investigation was termed *Polyporus rhinocerus *Cooke (Aphyllophoromycetideae), which is actually synonym of* Lignosus rhinocerus*. They found that the hot water extract of the sclerotia significantly reduced the growth of leukemic cell lines and Sarcoma S-180, but, surprisingly, no effect on the breast cancer cell line was revealed [8]. It is noted that Lai et al. [7] obtained their samples from a mycological institute in China, and, in their report, there was no mention of method used to confirm the identity of the mushroom. Our observations showed that morphological identification of tiger milk mushroom can be erroneous as there are several species of the mushrooms from the same family that are similar morphologically (unpublished observation). In this paper, we report our investigation of the antiproliferative activity of cultivar of *L. rhinocerus*, positively identified by genetic marker [9], on human breast and lung cancer cell lines and its cytotoxicity on normal breast and lung cell lines. 

## 2. Materials and Methods

### 2.1. Materials

Sclerotia from a *L. rhinocerus *cultivar (TM02, supplied by Ligno Biotech, Selangor, Malaysia) which have been positively identified by their internal transcribed spacer (ITS) regions of the ribosomal RNA [9] were freeze-dried and milled into powder using 0.2 mm sieve to yield a light brown, dry fluffy powder with milk-like taste. Four cell lines were used in this study: MCF-7, the human breast carcinoma cell line; A549, the human lung carcinoma cell line; 184B5, the normal human breast cell line; NL 20, the normal human lung cell line. All four cell lines were purchased from American Type Culture Collection (ATCC) and were cultured with different media: RPMI-1640 (Lonza, USA) for MCF-7 and A549 cell lines, MEGM Mammary Epithelial Cell Growth Medium (MEGM Bullet kit) (Lonza, USA) for 184B5 cell line, and Ham's F12 Medium (Lonza, USA) for NL 20 cell line. Growth media were enriched with 10% fetal bovine serum (FBS) (Sigma-Aldrich, USA) and supplemented with L-glutamine. Cells were grown in a humidified air with 5% CO_2_ at 37°C. All other chemicals used were of analytical grade.

### 2.2. Preparation of Cold Water Extract of *L. rhinocerus* Sclerotial Powder

10 g of *L. rhinocerus *sclerotial powder was dissolved in 100 mL of the milli-Q water with continual stirring for 24 h at 4°C. The mixture was then centrifuged at 2,500 ×g, and the supernatant was collected and freeze-dried.

### 2.3. Determination of Total Carbohydrate and Protein Content

Total carbohydrate content was determined by phenol-sulphuric acid method as described by Dubois et al. [10] using D-glucose as a standard. Protein concentration was determined by the Bradford method [11] using bovine serum albumin (BSA) as standard.

### 2.4. Cytotoxicity Assay

The cytotoxic activity of *L. rhinocerus *cold water extract was determined by MTT (3,(4,5-dimethythiazol-2-yl)-2,3-diphenyl tetrazolium bromide) method. This assay was carried out according to the method described by Ahn et al. [12] with slight modifications. The cells with optimal cell density were seeded in 96-well plate and incubated overnight for attachment. Different concentrations of the extracts were then added. After 72 hr incubation, MTT solution was added and incubated for 4 hr. All the solutions were then aspirated, and isopropanol was added to solubilize the formazan crystal. The absorbance was then determined by a microplate reader (Bio-Rad, USA) at 595 nm. Each measurement was performed in triplicates. The half-maximal inhibitory concentration (IC_50_) value was determined from the percentage of the cell viability versus final concentration of the extract curve.

### 2.5. DNA Fragmentation Studies

DNA fragmentation was used to investigate the mode of cell death induced by the *L. rhinocerus *cold water extract. Cells treated with IC_50_ dose of* L. rhinocerus *extract for 72 hr were harvested and lysed in a lysis buffer containing 1 M Tris-HCI, 0.5 M EDTA, Triton-X, and distilled water. The DNA was extracted by phenol : chloroform : isoamyl (25 : 24 : 1) mixture and precipitated with equal volume of ice-cold isopropanol. The DNA pellet was then dissolved in appropriate volume of RNase solution (10 mg/mL RNase I) and incubated at 37°C for 30 min. DNA was electrophorized on a 1.2% agarose gel containing GelRed. Finally, the apoptotic DNA fragments were visualized under a UV transilluminator and photographed [13].

### 2.6. Fractionation of *L. rhinocerus* Extract

The cold water extract of *L. rhinocerus *sclerotia was fractionated by Sephadex G-50 gel filtration column (2.6 × 40 cm) pre-equilibrated with 0.05 M ammonium acetate buffer. Elution was carried out at a flow rate of 2 mL/min using 0.05 M ammonium acetate buffer. Carbohydrate and protein content of each fraction was determined. The cytotoxicity (against MCF-7 and A549 cell lines) of the high-molecular-weight and low-molecular-weight fractions was examined by MTT method.

## 3. Results

### 3.1. Carbohydrate and Protein Contents

The cold water extract of *L. rhinocerus *sclerotia (termed LR-CW) was freeze-dried to yield a brownish powder with a yield of about 10% dry weight of the sclerotia powder. The carbohydrate and protein contents were determined to be 75% and 1.2%, respectively, of the dry weight of LR-CW.

### 3.2. Cytotoxic Activity of Cold Water Extract of *L. rhinocerus*

**Figure 1:**
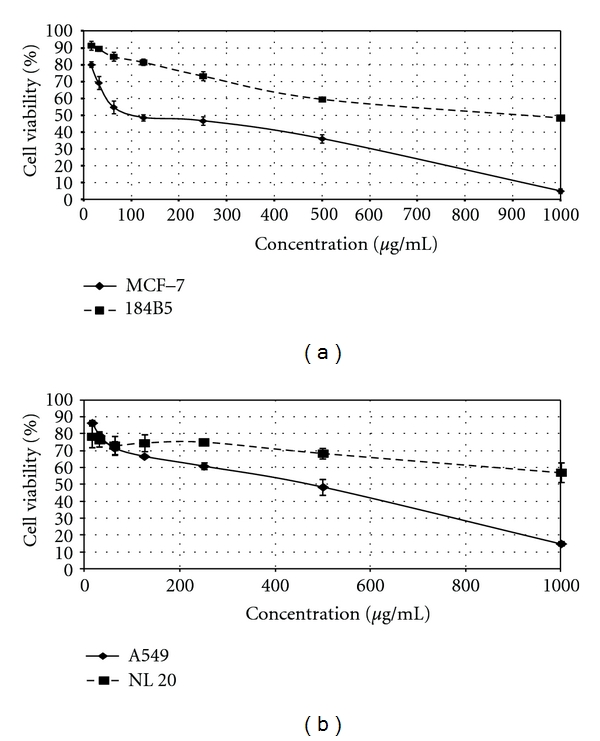
Antiproliferative activities of the cold water extract of *Lignosus rhinocerus* sclerotia (LR-CW). Human cancer cells (MCF-7 and A549) and human normal cells (184B5 and NL 20) were treated with different concentrations of LR-CWs ranging from 15.6 *μ*g/mL to 1000 *μ*g/mL for 72 hr, IC_50_ value was determined from the curve. (a) Cytotoxic activity of LR-CW against MCF-7 (solid line) and 184B5 (broken line). (b) Cytotoxic activity of LR-CW against A549 (solid line) and NL20 (broken line). Data was expressed as mean ± SD (*n* = 3).

Figure 1 shows the effect of different concentrations of LR-CW on the cell viability of both human breast and lung cancer cells as well as the human normal breast and lung cells. The cold water extract of *L. rhinocerus *sclerotia exhibited antiproliferative activity against both MCF-7 and A549 cells, with IC_50_ of 96.7 *μ*g/mL and 466.7 *μ*g/mL, respectively. In comparison, LR-CW did not show significant cytotoxic effect on human normal breast and lung cell lines, with IC_50_ values >900 *μ*g/mL (Table 1).

### 3.3. DNA Fragmentation Studies

**Figure 2:**
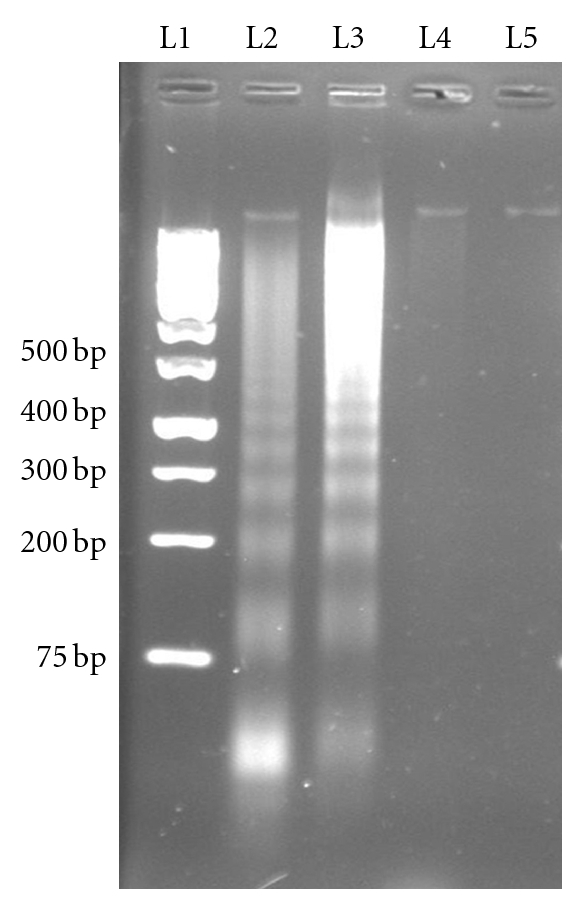
DNA fragmentation in cells treated with cold water extract of *Lignosus rhinocerus* sclerotia (LR-CW). Lane 1: 1 kb DNA ladder. Lane 2: LR-CW-treated MCF-7 cells; Lane 3: LR-CW-treated A549 cells. Lane 4: untreated MCF-7 cells. Lane 5: untreated A549 cells.

DNA fragmentation studies were used to examine whether the LR-CW induced apoptosis.  Figure 2 shows the presence of DNA ladder fragments on the LR-CW-treated MCF-7 and A549 cells, indicating that cell death was mediated by apoptosis [14]. A single band was observed on the untreated (control) cells, and there was no DNA ladder formation.

### 3.4. Fractionation of Cold Water Extract of *L. rhinoceros* (LR-CW)

**Figure 3:**
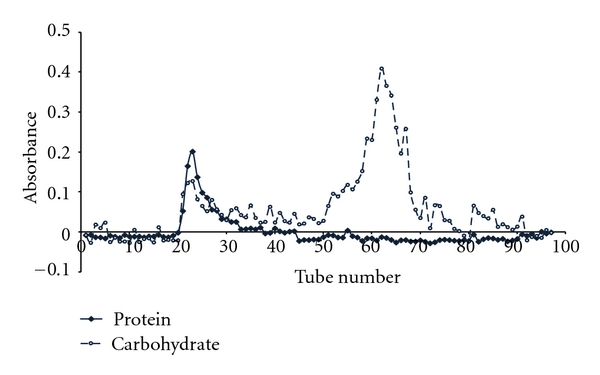
*L. rhinocerus* cold water extract (50 mg in 5 mL) was fractionated by Sephadex G-50 gel filtration chromatography. The column (2.6 × 40 cm) was pre-equilibrated with 0.05 M ammonium acetate buffer. Elution was carried out at a flow rate of 2 mL/min, and fractions of 3.5 mL were collected. The protein content (-♦-♦-) was determined by Bradford protein assay (absorbance at 595 nm), and the carbohydrate content (- ○ - - ○ - -) was determined by phenol-sulphuric method (absorbance at 490 nm).

**Figure 4:**
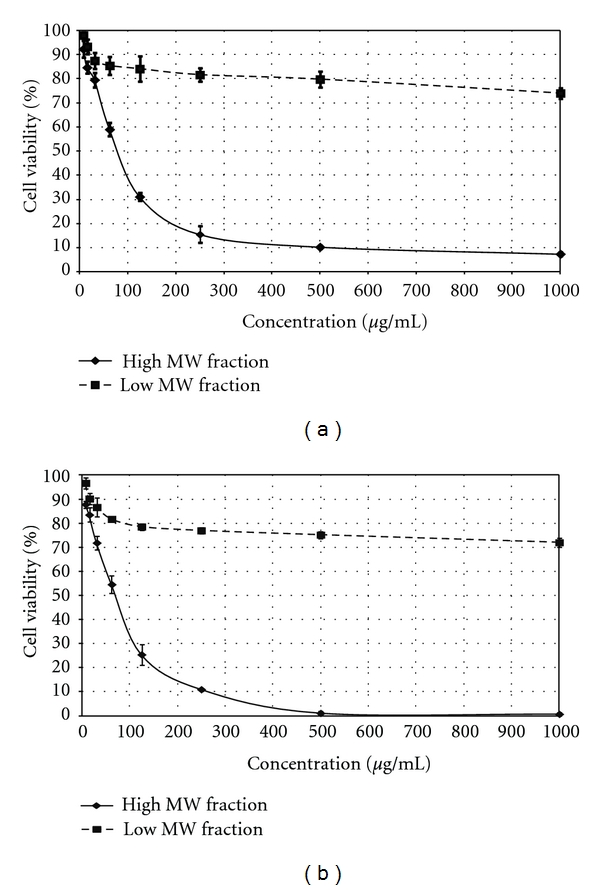
Antiproliferative activities of high- and low-molecular-weight fractions from cold-water extract of *Lignosus rhinocerus* sclerotia (LR-CW). Cells were treated with different concentrations of the fractions ranging from 7.8 *μ*g/mL to 1000 *μ*g/mL. (a) Top: antiproliferative activities of high- and low-molecular-weight fractions (solid line and broken line, resp.) against A549 cell line. (b) Bottom: antiproliferative activities of high- and low-molecular-weight fractions (solid line and broken line, resp.) against MCF-7 cell line. Data was expressed as mean ± SD (*n* = 3).

Fractionation of LR-CW by Sephadex G-50 gel filtration chromatography yielded two major peaks (Figure 3). The high-molecular-weight peak contained both protein (3.6%) and carbohydrate (68.7%), while the low-molecular-weight peak contained carbohydrate (31.8%) and very small amount of protein (0.15%) and other unidentified substances. The cytotoxicity of these two fractions was examined using human cancer cell lines MCF-7 and A549.  Figure 4 shows that the high-molecular-weight fraction exhibited strong antiproliferative activity against both MCF-7 and A549 cell lines, with IC_50_ of 70.0 *μ*g/mL and 76.7 *μ*g/mL, respectively. The low-molecular-weight fraction did not exhibit significant cytotoxicity, with IC_50_ > 1000 *μ*g/mL.

## 4. Discussion

The cold water extract of the *L. rhinocerus *cultivar contains mainly carbohydrate and a rather small amount of protein. This is similar to the composition of the cold alkaline extract of the sclerotia (82.3% carbohydrate and 1.3% protein) but very different from the composition of the hot water extract, which contains almost equal amount of carbohydrate and protein (37.4% and 41.3%, resp.) [7]. Apparently, the higher temperature used in the hot water extract managed to solubilize large amount of cellular structural/storage proteins. Fractionation of the LR-CW by Sephadex G-50 gel filtration yielded two fractions, the high-molecular-weight fraction that emerged at the void volume (Mol Wt > 30,000) and the low-molecular-weight fraction that emerged at the bed volume, representing a mixture of small molecules. Both high- and low-molecular-weight fractions contain carbohydrate, but protein was found only in the high-molecular-weight fraction. The high-molecular-weight fraction may contain polysaccharide-protein complex, as suggested by Lai et al. [7].

Our results indicated that LR-CW exhibited significant antiproliferative activity against the breast cancer cell MCF-7 and lung cancer cell A549. The antiproliferative action against MCF-7 cells provides a plausible scientific basis for the traditional use of *L. rhinocerus *sclerotia in breast cancer treatment by the Malaysian natives. Earlier, Lai et al. [7] reported that the hot water extract of *P. rhinocerus* (synonym of *L. rhinocerus*) exhibited antiproliferative activity against different kinds of leukemic cells (with IC_50_ ranging from 100 *μ*g/mL and 400 *μ*g/mL), but not MCF-7 [8]. Wong et al. [15] also reported that the cold alkaline extract of the mushroom sclerotia could stimulate human innate immune cells. Thus, it appears that *L. rhinocerus *sclerotia contain various antiproliferative agents with different actions and cell specificity. While the substance in the cold alkaline extract that potentiates immune response is likely the *β*-glucan [15], the nature of the antiproliferative in the cold and hot water extract is yet to be elucidated.

Our results also showed that the LR-CW was essentially not cytotoxic against the normal breast and lung cells (184B5 and NL 20, resp.). This is the first report to demonstrate that *L. rhinocerus *extract is not toxic to normal cells. The basis of the selective antiproliferative effect of LR-CW that appears to target cancer cells specifically is yet to be elucidated.

To further explore the nature of the antiproliferative agent in the LR-CW, the extract was fractionated by Sephadex G-50 gel filtration column into two fractions: high-and low-molecular-weight fractions. Only the high-molecular-weight fraction exhibited rather strong antiproliferative activity against the two cancer cells tested. The low-molecular-weight fraction was devoid of antiproliferative activity. As the high-molecular-weight fraction contains both carbohydrate and protein, the antiproliferative agent may either be a type of protein-carbohydrate complex and proteins that target metabolic processes or signal transduction in cancer cells. It is known that proteins such as proteases, ribosome inactivating proteins, and lectins could exert direct cytotoxic effects on cancer cells [2]. Further work is in progress to identify the nature of the cytotoxic agent in the high-molecular-weight fraction.

It is believed that the cytotoxic action of many anticancer agents is mediated by apoptosis [16]. Earlier, Lai et al. [7] showed that the antiproliferative effect of the hot water extract of *P. rhinocerus *against HL-60 cells was mediated by cell cycles arrest at G_1_ phase that led to apoptosis. Our DNA fragmentation studies clearly suggested that LR-CW kills MCF-7 and A549 cells also by inducing apoptosis. However, the apoptosis-inducing agent in LR-CW may not be the same as the one in hot water extract of *P. rhinocerus*, as the extracts differ in selectivity of cytotoxic action (see above).

## 5. Conclusion

The cold water extract of the sclerotia of the cultivated *L. rhinocerus *possesses cytotoxicity against breast and lung cancer cells but were non-toxic to the corresponding normal cells. Its cytotoxicity action is due to a high-molecular-weight fraction isolated from the cold water extract and that the cytotoxic action is mediated via apoptosis.

## Figures and Tables

**Table 1:** Cytotoxic activities (IC_50_) of cold water extract of sclerotia of *Lignosus rhinoceros* and the partial purified fractions of the extract.

Extracts or fractions	MCF-7 (*μ*g/mL)	A549 (*μ*g/mL)	184B5 (*μ*g/mL)	NL20 (*μ*g/mL)
LR-CW	96.7 ± 14.5	466.7 ± 43.7	906.7 ± 26.7	>1000
High-molecular-weight fraction	70.0 ± 5.8	76.7 ± 3.3	ND	ND
Low-molecular-weight fraction	>1000	>1000	ND	ND

Cells were treated with the cold water extract of sclerotia of *L. rhinocerus* (LR-CW) as well as the two fractions isolated from LR-CW. MCF-7 and A549 are human breast and lung cancer cells, respectively, whereas 184B5 and NL20 are normal human breast and lung cells, respectively. Treatment time was 72 hr. Results expressed as mean ± SEM (*n* = 3). ND: not determined.
